# Microparticles Release by Adipocytes Act as “Find-Me” Signals to Promote Macrophage Migration

**DOI:** 10.1371/journal.pone.0123110

**Published:** 2015-04-07

**Authors:** Akiko Eguchi, Anny Mulya, Milos Lazic, Deepa Radhakrishnan, Michael P. Berk, Davide Povero, Agnieszka Gornicka, Ariel E. Feldstein

**Affiliations:** 1 Department of Pediatrics, University of California San Diego (UCSD), La Jolla, California, United States of America; 2 Department of Cell Biology, Lerner Research Institute, Cleveland Clinic, Cleveland, Ohio, United States of America; Tohoku University, JAPAN

## Abstract

Macrophage infiltration of adipose tissue during weight gain is a central event leading to the metabolic complications of obesity. However, what are the mechanisms attracting professional phagocytes to obese adipose tissue remains poorly understood. Here, we demonstrate that adipocyte-derived microparticles (MPs) are critical “find-me” signals for recruitment of monocytes and macrophages. Supernatants from stressed adipocytes stimulated the attraction of monocyte cells and primary macrophages. The activation of caspase 3 was required for release of these signals. Adipocytes exposed to saturated fatty acids showed marked release of MPs into the supernatant while common genetic mouse models of obesity demonstrate high levels of circulating adipocyte-derived MPs. The release of MPs was highly regulated and dependent on caspase 3 and Rho-associated kinase. Further analysis identified these MPs as a central chemoattractant in vitro and in vivo. In addition, intravenously transplanting circulating MPs from the ob/ob mice lead to activation of monocytes in circulation and adipose tissue of the wild type mice. These data identify adipocyte-derived MPs as novel “find me” signals that contributes to macrophage infiltration associated with obesity.

## Introduction

Obesity has reached epidemic proportions in most of the western world. With obesity comes a variety of adverse health outcome such as dyslipidemia, hypertension, glucose intolerance, and hepatic steatosis which are grouped into the so-call metabolic syndrome [1–3]. Insulin resistance is a common central feature of this syndrome [4–6]. The adipose tissue has emerged as an important player in this process. The expanding adipose tissue during weight gain is associated with hypertrophy of adipocytes, macrophage infiltration, and increase production of a variety of bioactive substances such as cytokines, chemokines, and reactive oxygen species (ROS) [7–11]. Indeed, in both humans and rodents macrophages accumulate in adipose tissue with increasing body weight and preventing the accumulation of macrophages protects against the multiple obesity related metabolic complications [12–15].

The pathogenic mechanisms resulting in macrophage recruitment to adipose tissue are under intense investigation and remain incompletely understood. Increased production and release of certain chemokines potentially as a result of local hypoxia in an expanding adipose tissue bed has been implicated [16–18]. The vast majority of infiltrating macrophages in both adipose tissue from obese mice and humans are found around death or dying adipocytes forming characteristic morphological structures called “crown-like structures” [8, 19–22]. This lead us to hypothesize that stressed adipocytes release “find me” signals that attract professional scavenger cells to engulf and digest them. Here, we present evidence that hypertrophied and stressed adipocytes secrete chemotactic signals that induce macrophage migration in a caspase 3 dependent manner. This attraction was independent of previously described “find me” signals released by cancer cells and thymocytes including nucleotides and the lipid lysophosphatidylcholine (LPC) [23, 24] and only partially the result of production by adipocytes of the chemokine monocyte chemoattractant protein-1 or monocyte chemotactic protein-1 (MCP-1). Through several lines of evidence, we further identify adipocyte-derived MPs, small membrane-bound particles released from dying or activated cells that have been linked to important roles in cell-cell interaction, immune regulation and tissue regeneration, as novel chemotactic factors in a process involving caspase 3 and Rho-associated kinase activation. These findings identify a novel mechanisms for dying or stressed cells to secrete “find me” signals and give important clues of how macrophages infiltrate obese adipose tissue.

## Materials and Methods

### Animals studies

These experimental protocols were approved by the Institutional Animal Care and Use Committee at the University of California, San Diego and Cleveland Clinic. All efforts were made to minimize pain and distress during animal husbandry and experimental assessments. In order to study the spectrum of human obesity, leptin-deficient (ob/ob)) mice were used. Leptin-deficient (ob/ob) and ob-control mice (n = 5 in each group), age 12 weeks, were purchased from Jackson Laboratory. For MP transplantation experiment, Leptin-deficient ob/ob and ob-control, age 10–11 weeks were purchased from Jackson Laboratory.

### In-vitro cell culture studies

Mouse 3T3-L1 adipocytes (American Type Culture Collection (ATCC), Manassas, VA) were grown and maintained at no higher than 70% confluence in Dulbecco’s Modified Eagle Medium (Gibco, Camarillo, CA) supplemented with 10% fetal bovine serum (Cellgro, Manassas, VA), Penicillin and Streptomycin (growth medium) at 37°C in a 10% CO_2_ incubator. Medium was replaced every other day until cells reach confluence. For differentiation into mature 3T3-L1 adipocytes, cells were grown 2-day post confluence in growth medium and then the cells were induced to differentiate in growth medium supplemented with insulin, 3-isobutyl-1-methylxanthine and dexamethasone (Cayman Chemical, Ann Arbor, MI, USA) as described previously [25]. Three days post induction, medium was replaced with insulin only medium (growth medium supplemented with only insulin) for additional five to seven days, medium was replaced every other day during this period and accumulation of lipid droplet was monitored under microscope. At least 95% of the cells showed an adipocyte phenotype at the end of differentiation period. For MPs generation, differentiated 3T3-L1 adipocytes were treated in the absence or presence of various concentrations of free fatty acids (palmitic acid) complex with 1% bovine serum albumin (BSA, free fatty acid free, endotoxin free) (Sigma Chemical Co., St. Louis, MO, USA) for up to 24 hours. In order to determine the effect of Rho-associated kinase or caspase-3 effect, 3T3L1 differentiated adipocytes were stimulated with above stimuli in the absence or presence of Rho-associated kinase inhibitors, Y27632 or fasudil (HA-1077) (Sigma Chemicals Co., St. Louis, MO, USA) or a selective caspase-3 inhibitors, Ac-DEVD-CHO (BD Pharmingen). In some experiments, 3T3L1 adipocytes were treated with stimuli above in the absence or presence of 0.025 U/ml apyrase (New England Biolabs (Ipswich, MA), 0.5 U/ml phospholipase-D (Calbiochem, La Jolla, CA) or 50 μg/ml control IgG or MCP-1 antibody (Biolegend, San Diego, CA) to determine whether nucleotides, lysophosphatidylcholine or MCP-1, respectively, were the chemotactic factors released by stressed adipocytes. 3T3-L1 cells (undifferentiated) were treated for 24 h in the 0.5 mM palmitic acid complex with 1% bovine serum albumin or only 1% bovine serum albumin as a control.

Mouse primary adipocytes were isolated from epididymal AT of ob/ob mouse with modification of described method [26]. After filtration of collagenized AT through a 250-μm mesh, adipocytes were separated, washed twice by centrifugation at 50 g for 5 min at room temperature, and cultured in DMEM/F12 Medium supplemented with 2 mM L-Glutamine and Penicillin/Streptomycin at 37°C in a 5% CO_2_ incubator. For MP generation, cells were treated for 40 h in the 1 mM palmitic acid complex with 1% bovine serum albumin or only 1% bovine serum albumin as a control. Isolated stromal vascular fraction (SVF) was cultured in DMEM/F12 Medium supplemented with 2 mM L-Glutamine and Penicillin/Streptomycin at 37°C in a 5% CO_2_ incubator. They treated for 24 h in the 0.5 mM palmitic acid complex with 1% bovine serum albumin or only 1% bovine serum albumin as a control.

RAW264.7 monocyte / macrophages cell line was obtained from ATCC and cultured in DMEM medium supplemented with 10% FBS and Penicillin/Streptomycin in a 37°C, 5% CO_2_ incubator. The cells were passage twice a week.

### Measurement of cell death/apoptosis

Caspase 3/7 activity assay was measured using the ApoOne Homogeneous Caspase 3/7 Assay (Promega, Madison, WI) according to manufacturer’s instruction. Briefly, differentiated 3T3-L1 adipocytes were trypsinized and diluted to 2x10^5^ cells/ml and 20,000 cells were plated to each well of 96 well plate. Cells were incubated for 2 hours before treatment for adhesion. Cells were then treated either with or without free fatty acids (FFA) complex in the absence or presence of selective caspase-3 inhibitor for additional 4 hours. At the end of incubation, caspase 3/7 activities were assayed following the protocol described by the manufacturer and estimated from the fluorescence at the excitation wavelength of 485 nm and the emission wavelength of 538 nm using the Spectra Max Gemini EM (Molecular Devices, Sunnyvale, CA). DMEM medium mixed with the same volume of ApoOne Homogeneous Caspase 3/7 reagent was used as blank. The release of lactate dehydrogenase (LDH) assay in cell culture medium was measured using CytoTox 96_ Non-Radioactive Cytotoxicity Assay (Promega) according to manufacture’s instruction.

### Cell-derived MPs isolation

Conditioned media of mature differentiated 3T3-L1 adipocytes post stimulation were collected and cleared from cells and cell debris by centrifugation at 2000xg for 10 minutes, 10°C. Supernatant were immediately processed for MPs isolation by ultracentrifugation at 100,000xg for 60 minutes, 10°C using an ultracentrifuge (Beckman L7-65, Beckman-Coulter, Palo Alto, CA) [27]. Post ultracentrifugation, supernatant was removed and pellet was resuspended in 200–500 μl of 1x PBS or DMEM medium (no serum), respectively and kept at 4°C until further use (maximum time for storage was 1 week).

### Circulating MP isolation

Whole mouse blood was collected by cardiac puncture of leptin-deficient (ob/ob) mice into tubes containing anticoagulant under anesthesia via i.p. injection using a 21G needle and a mixture of 100 mg/Kg of Ketamine and 10 mg/Kg of Xylazine dissolved in a 0.9% saline solution with euthanasia carried out by carbon dioxide exposure. Circulating MP was isolated with modification of described methods [27] [28]. Blood was centrifuged at 1,200 g for 15 min and 12,000 g for 12 min at 22°C to obtain platelet free plasma (PFP). The MPs present in PFP were then analyzed by flow cytometry. For Western blot circulating MPs were isolated by additional ultracentrifuge spin at 20,000 g for 30 min at 10°C. To extract MP for transplantation experiment, PFP was obtained from ob/ob or ob control mice and MPs were isolated by 10–70% sucrose gradient at 100,000 g for 16 h at 10°C following washing MPs fraction at 100,000 g for 60 min at 10°C. Purified MPs were suspended in PBS and kept at -80°C.

### Flow cytometry analysis of MPs

MPs resuspended in PBS (50 μl) or 30 to 50 μl of PFP were incubated in the dark for 30 minutes at room temperature with or without 5 μl of Alexa Fluor 488-conjugated Annexin V (Molecular Probe, Eugene, OR). MPs acquisition was performed by means of the BD LSRII Flow Cytometer System (BD Biosciences, San Jose, CA) and the data was analyzed using FlowJo software (TreeStar Inc., Ashland, OR). Gating parameters were defined using 1-μm latex beads (Sigma Chemicals Co.) and negative control. MPs were identified using a forward-scatter analysis. The MP number was counted using 2.5-μm Alignflow alignment beads (Molecular Probe, Eugene, OR) as the size standards.

### MP size determination

MPs were isolated from 3T3L1 or plasma described above and suspend in 1x PBS. For dynamic light scattering analysis, entire size was measured by Zetasizer nano ZS90 (Malvern). For Transmission electron microscope, MPs were adhered to 100 mesh Formvar and carbon coated grids for 5 minutes at room temperature. Grids were washed once with water, stained with 1% uranyl acetate (Ladd Research Industries, Williston VT) for 1 minute, dried and viewed using a JEOL 1200 EXII transmission electron microscope. Images were captured using a Gatan Orius 600 digital camera (Gatan, Pleasanton CA).

### Western blot analysis of MPs and adipose tissue

MPs was isolated from conditioned medium of 3T3L1 adipocytes treated with 0.5 mM palmitic acid as described above. The pelleted MPs was then resuspended in 200 μl of 1x PBS buffer and 50 μl of it was resolved by 8% sodium dodecyl sulfate polyacrylamide gel electrophoresis (SDS-PAGE), transferred to polyvinylidene difluoride (PVDF) membrane (Biorad, Hercules, CA) and blocking for at least 1 h with 5% bovine serum albumin in 1x PBS, 0.1% Tween 20 (PBS-T). Blots were then hybridized using antibody specific for fatty acid binding protein 4 (FABP4), MCP-1 (Cell Signaling, Danvers, MA), chemerin (R&D Systems), adipocyte complement-related protein of 30 kDa (Acrp30, adiponectin) (Alpha Diagnostic International, San Antonio, TX), perilipin A (Fitzgerald, Concord, MA, or Abcam, Cambridge, MA) and visfatin (Biovision, Mountain View, CA). Protein was visualized by SuperSignal West Pico chemiluminescence substrate (Pierce biotechnology, Rockford, IL). Band intensity was analyzed using image J software (National Institutes of Health, Bethesda, MD).

Epididymal adipose tissues (300 mg) were homogenized using a polytron on ice in 1.5 ml of lysis buffer (20 mM Tris, pH 7.5, 150 mM NaCl, 1 mM EDTA, 1 mM EGTA, 1% NP40, protease inhibitor cocktail (Roche Applied Science, Mannheim, Germany), and 1 mM sodium vanadate). Adipose tissue lysates were continuous rotated for 1 hour at 4°C, followed by centrifugation for 10 minutes at 14,000 g. The fat cake was removed from lysate and supernatant was transferred to the new eppendorf tube. Total protein was quantitated by Pierce 660 nM protein assay (Pierce Biotechnology). Adipose tissue lysate (50 μg) was resolved by 8% SDS-PAGE, transferred to PVDF membrane and probed for phospho-MYPT1 (Thr696) (Millipore, 1:1000), MYPT1/MYPT2 (C-term) (Epitomics, Burlingame, CA, 1:1000) and actin (Cell Signaling, 1:2000) protein using SuperSignal West Femto chemiluminescence substrate (Pierce Biotechnology). Band intensity was analyzed using image analysis software, ImageQuant TL software (GE Healthcare, Piscataway, NJ)

### Isolation of stromal vascular fraction

Stromal vascular fraction was isolated from epididymal AT of ob/ob mouse with modification of described method [26]. After filtered through a 250 μm mesh and centrifuged at 500 rpm for 5 min, stromal vascular fraction (SVF) cells were pelleted by centrifugation at 1,000 rpm for 10 min, incubated with erythrocyte-lysing buffer (eBioscience, San Diego, CA), and washed with PBS twice. SVF cells were suspended with 3% FBS-PBS and incubated with labeled antibodies, F4/80 (AbD Serotec, Raleigh, NC) or CD11c (eBioscience, San Diego, CA). Macrophage infiltration was analyzed by Flow Cytometer [Becton Dickinson, LSR II].

### Isolation of mouse peritoneal macrophages

C57BL/6 mice fed standard chow diet were injected intraperitoneally with 1 ml sterile 4% thioglycollate (Sigma Chemical Co.). After three days, mice were killed by carbon dioxide exposure, and subsequently peritoneal cells were collected by lavage. Cells were cultured in RPMI containing 10% FBS and Penicillin/Streptomycin for 2 hours to allow macrophages adhering to the plates. Non-adherent cells were removed by washing with warm 1x PBS, and the adherent macrophages were scraped in the presence of RPMI medium (no serum), and seeded onto 8-μm transwell insert for in vitro chemotaxis assay.

### In vitro chemotaxis assays

Migration of mouse peritoneal and RAW 264.7 macrophages was measured in a modified Boyden chamber migration assay using Transwell inserts with an 8 μm pore size membrane (Millipore, Billerica, MA). Supernatants of 3T3-L1 adipocytes, isolated MPs, MPs-free supernatants, or medium containing 10 ng/ml MCP-1 (R&D Systems, Minneapolis, MN) as positive control were placed in the lower chamber. Two hundred thousand macrophages were suspended in serum free DMEM or RPMI medium and loaded into the upper migration chamber. The cell migration were performed for 16 hours, the cells that had not migrated and remained in the upper chamber were removed by gently swiping the membrane with cotton tips. The cells that migrated were stained with 4’,6-diamidino-2-phenylindole (DAPI) and mounted on the cover glass. The amount of cells that migrated was quantified from 6 fields of 10x magnification/condition using fluorescence microscope.

### In vivo chemotaxis assays

Ultracentrifuge purified MPs from 3T3-L1 adipocytes treated with palmitic acid or control were intraperitoneally injected into 8 weeks old C57BL/6 mice. After three or four days, cells infiltrated in the peritoneal cavity were collected with 5 ml x 2 of cold PBS. After removing red cells with RBC lysis buffer, isolated cells were incubated with CD45 (BD Pharmingen), CD11b (eBioscience), F4/80 (AbD Serotec, Raleigh, NC), and Ly6G (clone 1A8) (Biolegend, San Diego, CA) for 30 min on the ice, washed, and analyzed by flow cytometry (BD LSRII Flow Cytometer System).

### In vivo MP transplantation assay

Hundred μg of purified circulating MPs from ob/ob and ob control animals resuspended in 250 μl PBS, or 250 μl PBS only (mock) was intravenously injected into 10 weeks old C57BL/6 mice. Mouse blood was collected by cardiac puncture in EDTA coated tube under anesthesia via i.p. injection using a 21G needle and a mixture of 100 mg/Kg of Ketamine and 10 mg/Kg of Xylazine dissolved in a 0.9% saline solution with euthanasia carried out by carbon dioxide exposure. White cells were separated by Hetasep (Stemcell technologies, Canada) and incubated with RBC lysis buffer. Cells infiltrated in epididymal adipose tissue were isolated by collagenase treatment using previously described method [26]. These cells were incubated with CD11b (eBioscience), Ly6C (eBioscience), CD204 (AbD Serotec), and CD45 (clone 30-F11) (BD Pharmingen) for 30 min on the ice, washed and analyzed by flow cytometry (BD LSRII Flow Cytometer System).

### Statistics

All data are expressed as mean ± S.D. unless otherwise indicated. Differences between groups were compared by analysis of variance (ANOVA) with subsequent application of Tukey’s multiple comparison and Student’s t-test, as appropriate. GraphPad software (La Jolla, CA) was used to perform all analysis and to construct all graphs.

## Results

### Adipocytes release factors that induced migration of monocyte cells and primary macrophages

**Fig 1:**
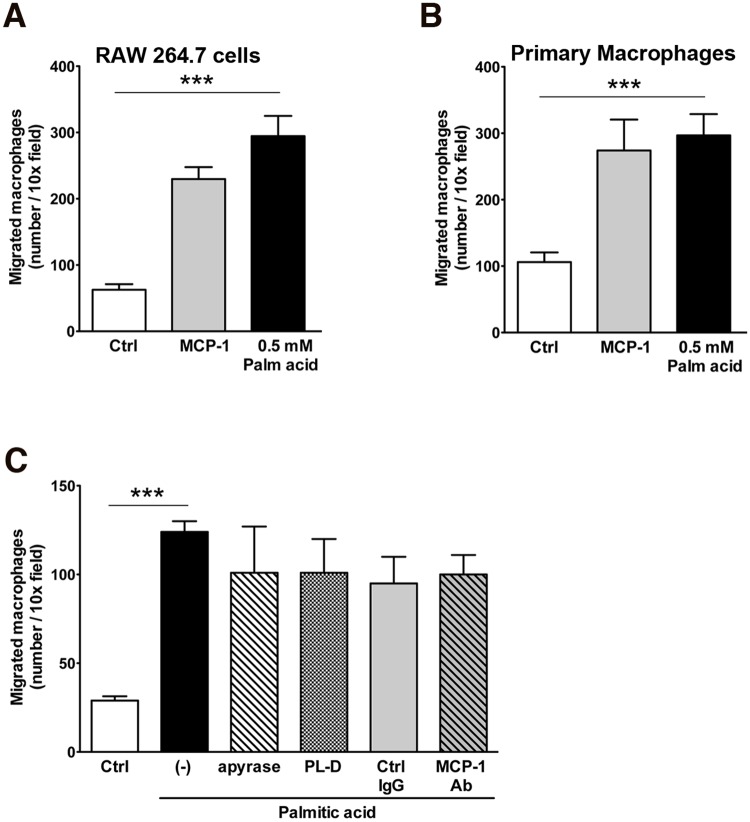
Attraction of monocytes and macrophages to supernatants of mature adipocytes exposed to palmitic acid. Migration of (A) RAW264.7 cells or (B) primary mouse macrophages through a transwell (8 μm pore size) to supernatants from untreated (control) or treated differentiated mature adipocytes with 0.5mM palmitic acid. MCP-1 (50 ng/ml) was used as positive control. (C) Adipocytes were treated with 0.5 mM palmitic acid in the absence or presence of 0.025 U/ml apyrase, 0.5 U/ml phospholipase-D or 50 μg/ml control IgG or MCP-1 neutralizing antibody. Macrophages that migrated to the lower chamber were stained with DAPI and the number of cells was counted under fluorescence microscopy. Values represent as mean ± S.D. of representative experiment. * P < 0.05; ** P < 0.01; ***P < 0.001 compared to controls.

It has long been speculated that in higher organisms where dying cells and phagocytes are not located in immediate proximity, dying cells secrete compounds that recruit monocyte and macrophages to the site of death. To determine whether stressed or dying adipocytes release such factors, we initially assessed cell-free supernatants of mature adipocytes exposed to the lipotoxic free fatty acids, palmitic acid, that are typically present in the obese adipose tissue for their ability to attract RAW 264.7 cells or primary mouse macrophages in transwell migration assays (Fig 1A and 1B). Supernatants from adipocytes treated with palmitic acid resulted in marked increase in monocyte migration which was more than 3-fold higher than controls and greater than the migration elicit by the potent chemotactic factor MCP-1 (Fig 1A and 1B). In order to determine the nature of the attraction signals release by adipocytes, we next access two previously described “find me” signals in other model systems including extracellular nucleotides, and the phospholipid LPC, as well as MCP-1, which has been proposed to contribute to macrophage infiltration into obese adipose tissue in vivo as potential candidates [29]. Treatment of supernatants from adipocytes with either apyrase or phospholipase D, enzymes that hydrolyse nucleotides or LPC respectively, as well as neutralizing anti-MCP-1 antibodies did not affect the chemotactic activity of palmitic acid treated adipocytes (Fig 1C).

### Caspase 3 activation is required for release of chemotactic factor by stressed adipocytes

**Fig 2:**
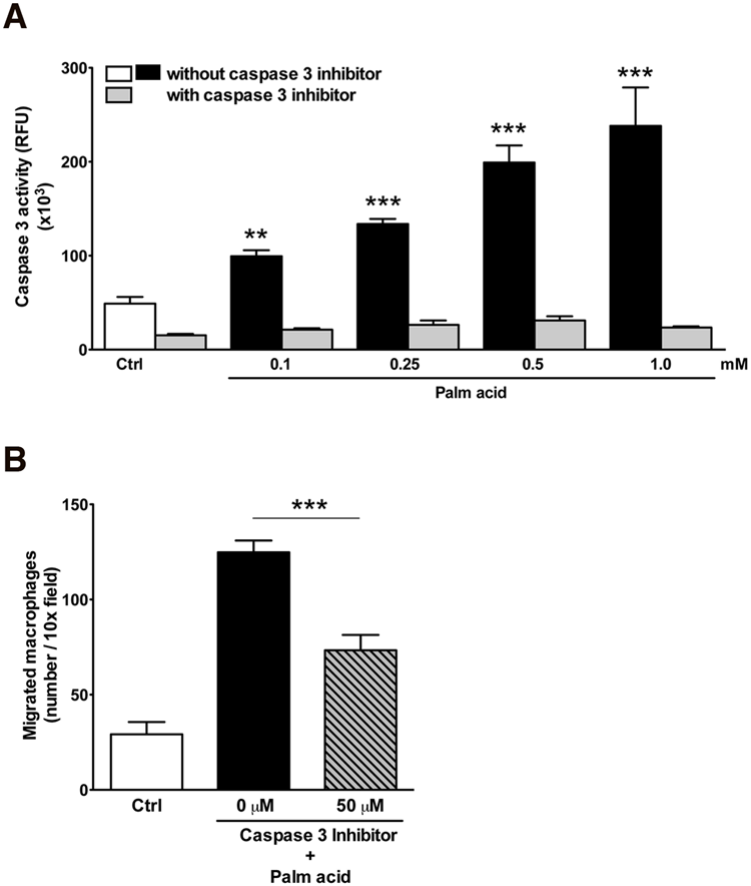
Caspase 3 activation is required for attraction of macrophages to stressed adipocytes. Caspase-3 activity assay in adipocytes treated with (A) a range of doses of palmitic acid (0.1 to 1 mM) in the absence or presence of a selective caspase 3 inhibitor (Ac-DEVD-CHO). Differentiated adipocytes were plated on black 96-well plate for 2 hrs followed by the different treatments for additional 4 hrs. Caspase-3 activity assay was determined by the Apo-One Homogeneous Caspase 3/7 fluorescent assay as described under Experimental Procedures. Assays were performed on three replicates for each treatment. Values represent as mean ± S.D. ** P < 0.01; ***P < 0.001 compared to controls. Transmigration of primary mouse macrophages to supernatants from differentiated adipocytes treated with (C) palmitic acid, in the absence or presence of increasing doses of the caspase-3 inhibitor was assessed. Values represent as mean ± S.D. ***P < 0.001 compared to stressor alone (no caspase inhibitor).

As the release of soluble attraction signals by cancer cells and thymocytes have been shown to depend on the activation of caspase 3, we next assessed whether exposure of mature adipocytes to palmitic acid results in caspase 3 activation. Indeed, we found marked, dose-dependent increase in caspase 3 activation in adipocytes exposed to this free fatty acid (Fig 2A). In order to determine whether adipocyte caspase activation is required for the production of chemotactic factors, adipocytes were co-incubated with palmitic acid in the presence or absence of a selective caspase 3 inhibitor. We found that the generation of attraction signals by palmitic acid treated adipocytes was indeed dependent on caspase activation as co-incubation with the caspase inhibitor abolished the migration activity (Fig 2B). Furthermore, the release of “find me” signals was not associated with changes in membrane integrity as measured by propidium iodide exclusion (S1A Fig) or with necrotic cells as measured by LDH release (S1B Fig), excluding leakage of cytoplasmic content and suggesting that the release of attraction signals by adipocytes is a regulated process. Thus, activation of caspase 3 appears to be crucial for the release of “find me” signals by adipocytes.

### Adipocyte-derived MPs are released during lipotoxic stress in a caspase 3 and Rho-associated kinase dependent manner

**Fig 3:**
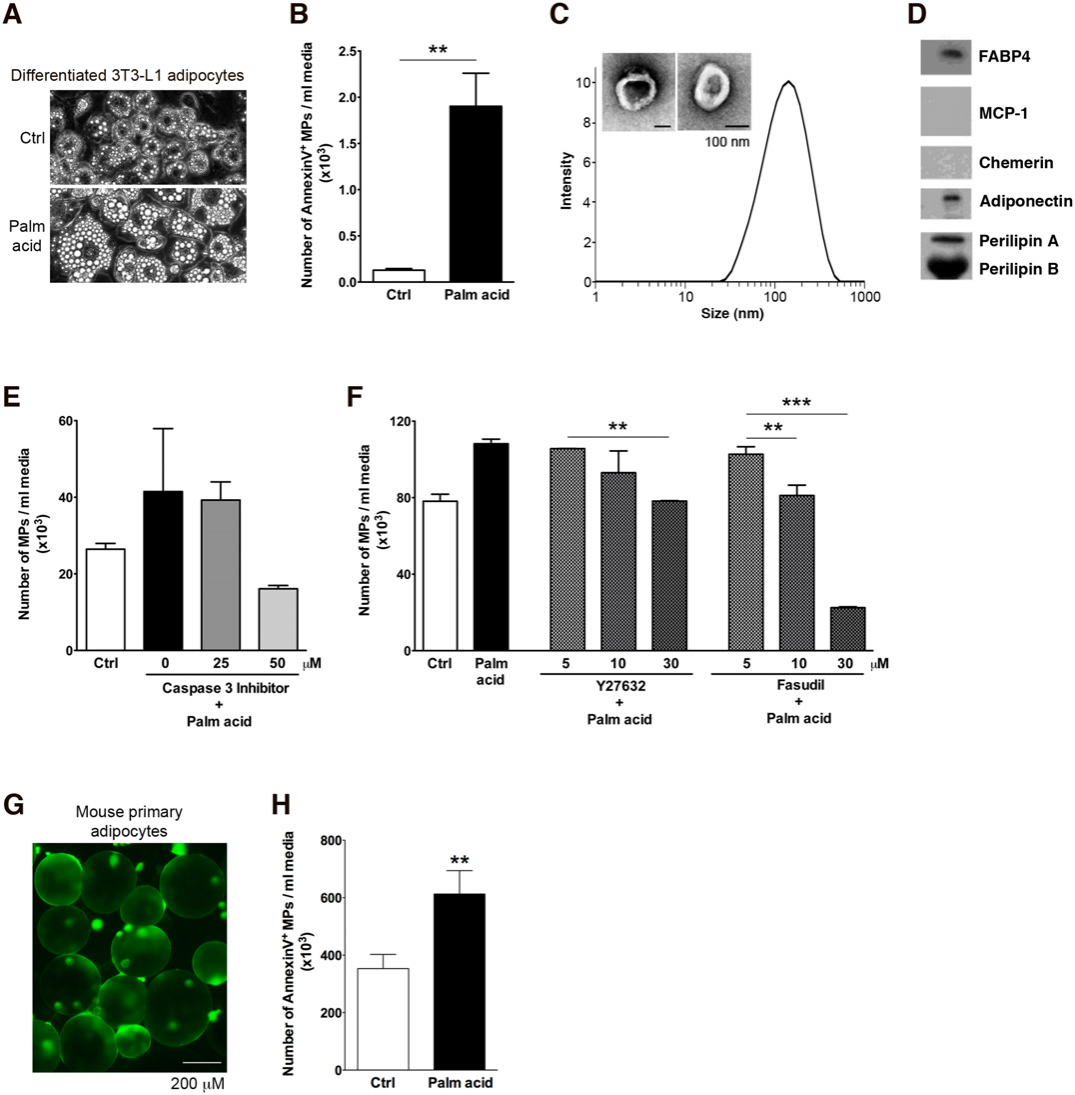
MPs are released by stressed adipocytes in a caspase 3 and Rho-associated kinase dependent manner. (A-D) Characterization of adipocyte-derived MPs. (A) Morphology of the differentiated 3T3-L1 adipocytes treated with control, 0.5 mM palmitic acid. (B) The number of Annexin V positive MPs was quantitated by flow cytometry. (C) Dynamic light scattering analysis and Transmission Electron Microscopy of isolated MPs. Isolated MPs were measured by Zetasizer and analyzed using intensity. (D) Western Blot analysis of MPs released by adipocytes. Isolated MPs were fractionated by SDS-PAGE and probed with FABP4, MCP-1, Chemerin, Adiponectin and Perilipin antibody. (E-F) Differentiated adipocytes were incubated with or without palmitic acid in the absence or presence of a selective caspase-3 inhibitor (E), or a range of doses of two different Rho associated kinase inhibitors (Y27632 and fasudil) (F) for up to 12 hrs. Supernatants were then collected and MPs isolated by ultracentrifugation as detailed in methods section. (G) Morphology of isolated mouse primary adipocytes. (H) Number of annexin V positive mouse primary adipocyte-derived MPs assessed by flow cytometry. Values represent mean ± S.D. * P < 0.5; ** P < 0.01; ***P < 0.001 compared to controls.

Our findings demonstrating the regulated release of attraction signals by stressed adipocytes occurring mostly through distinct mechanisms from the previously described “find me” signals led us to further explore the potential identity of alternative recruitment signals. Subsequent, several lines of evidence identified adipocyte-derived MPs as a possible key “find me” signal release by stressed adipocytes. Exposure of adipocyte to palmitic acid that induces chemoattractant activity resulted in hypertrophy of adipocytes (Fig 3A) and in marked production and release into the supernatants of annexin V positive MPs (Fig 3B). Notably, the production of annexin V positive MPs in 3T3-L1 cells (undifferentiated) was not increased (S2A Fig). To establish the characteristics of the adipocyte-derived MPs, we next performed a series of studies including dynamic light scattering analysis and transmission electron microscopy (Fig 3C). Adipocyte-derived MPs have a diameter ranging between 30 and 500 nm (mean diameter 142 nm for palmitic acid-treated adipocytes) corresponding to MP size [30] (Fig 3C). Moreover, western blot analysis of MPs from palmitic acid treated adipocytes expressed a variety of adipocyte-specific markers including FABP4, adiponectin, and perilipin A/B (Fig 3D), and none expressed MCP-1 (Fig 3D). The release of MPs was abolished by co-incubation with a selective caspase 3 inhibitor (Fig 3E), as well as by Rho-associated kinase inhibitors (Fig 3F). Furthermore, to investigate whether stressed primary adipocytes increase MP release, we isolated mouse primary adipocytes (Fig 3G) and SVF from epididymal AT of obese mice and treated them with palmitic acid. Here we demonstrated that the production of annexin V positive MPs was also significantly increased in palmitic acid-stressed mouse primary adipocytes as compared to control treatment (Fig 3H). On the other hand, the production of annexin V positive MPs were not increased in SVF with palmitic acid treatment compared to control treatment (S2B Fig).

### Adipocyte-derived MPs attracts macrophage in vitro and in vivo

**Fig 4:**
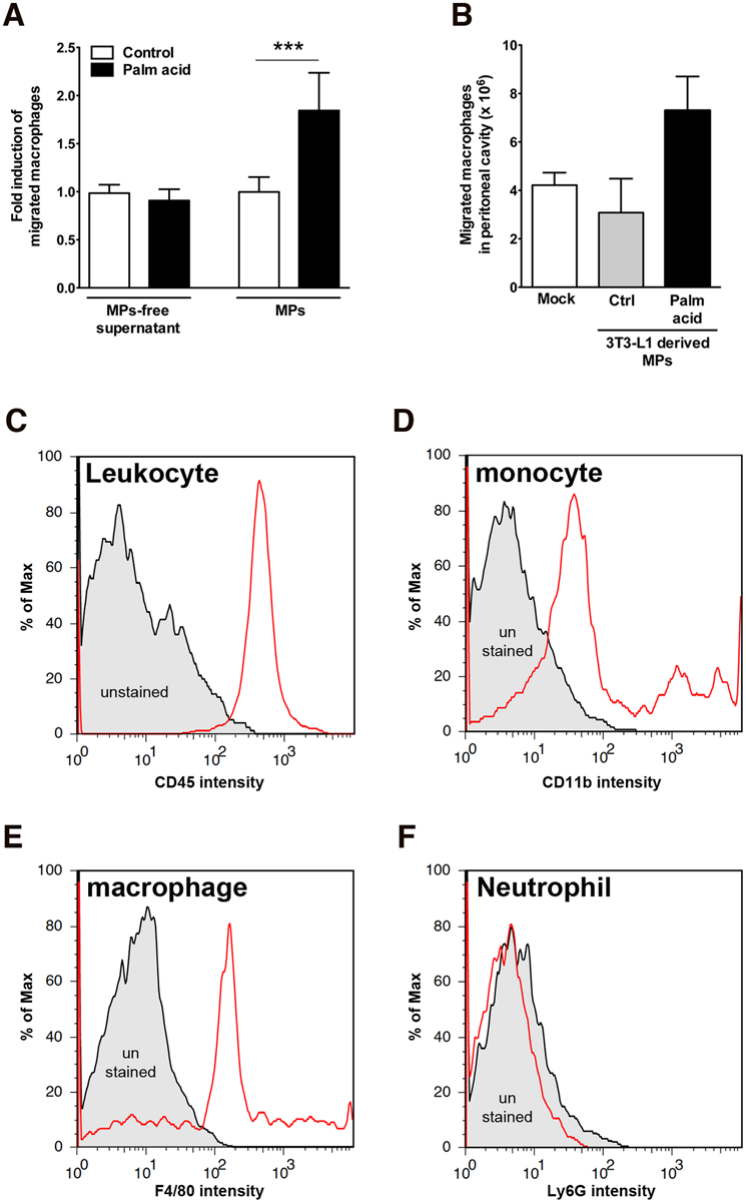
Adipocyte-derived MPs mediates attraction of macrophages in vitro and in vivo. (A) In vitro chemotaxis assay of MPs-free supernatant and MPs from adipocytes treated with palmitic acid. Values represent mean ± S.D. ***P < 0.001 compared to controls. (B-F) C57BL/6 mice were injected intraperitoneally with 1x10^6^ adipocyte-derived MPs (isolated from 3T3-L1 adipocytes treated with 0.5 mM palmitic acid or without palmitic acid) or controls (n = 3 each group). (B) Four days post injection infiltrated cells were isolated from peritoneal cavity by lavage and counted. (C-F) flow cytometry analysis of infiltrated cells. The infiltrated cells were stained by CD45 (leukocyte common antigen) (C), CD11b (monocytes) (D), F4/80 (macrophages) (E), or Ly6G (neutrophils) (F). Values represent mean ± S.E.M. (D) In vivo macrophages migration. C57BL/6 mice were injected intraperitoneally with 1x10^6^ palmitic acid-derived MPs or vehicle alone (n = 5 per group). Three days post injection; macrophages were isolated from peritoneal cavity by lavage. Number of macrophages present in peritoneal cavity was counted. Values represent mean ± S.E.M.

We next examined the effects on macrophages of purified adipocyte-derived MPs from control and stressed adipocytes and demonstrated that MPs formed by treatment of adipocytes with palmitic acid induce marked macrophage migration (Fig 4A). While supernatants of these cells depleted of MPs lost their chemotactic activity (Fig 4A). Next, to test whether adipocyte-derived MPs could induce macrophage migration in vivo C57BL/6 mice were injected intraperitoneally with cell-specific MPs from palmitic acid or control treated adipocytes. Injection of MPs derived from palmitic acid treated adipocytes resulted in an almost two-fold increase of infiltrated cells compared to control MPs or saline injection after four days post injection (Fig 4B). All infiltrated cells were leukocytes (CD45 LCA; white cell common antigen) (Fig 4C) and the main population of leukocytes was monocytes (CD11b positive) (Fig 4D) and macrophages (F4/80 positive) (Fig 4E), but not neutrophils (Ly6G negative) (Fig 4F).

### Circulating levels of MPs are increased in obesity

**Fig 5:**
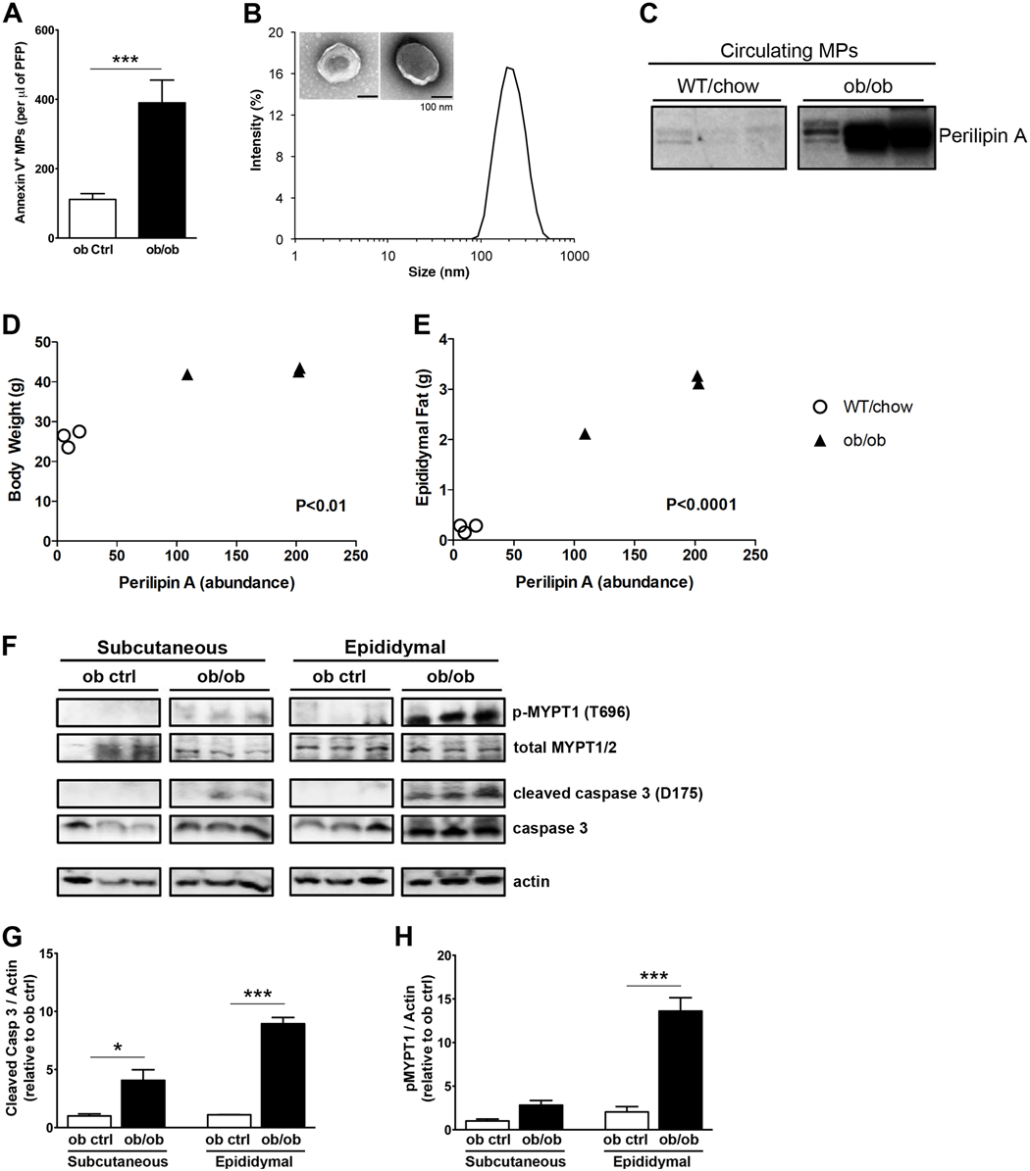
Obesity is associated with increased levels of circulating MPs and increased adipose activity of Rho associated kinase and caspase 3 Annexin V positive MPs from (A) ob control or ob/ob mice. Blood was collected by cardiac puncture and PFP was obtained as detailed in methods section. Annexin V positive MPs were analyzed by flow cytometry (n = 5 each group). (B) Dynamic light scattering analysis and Transmission Electron Microscopy of isolated circulating MPs. (C) Western blot analysis of perilipin A levels in MPs isolated from mouse PFP in WT mice on a regular chow diet and ob/ob mice (n = 3 each group). Perilipin A abundance in MPs isolated from mouse PFP correlated to: (D) total mouse body weights and (E) weight of extracted mouse epididymal fat pads. (F) Western blots of p-MYPT1, total MYPT1/2, cleaved (active) caspase 3, total caspase 3, and actin levels in subcutaneous and epididymal adipose tissue lysates from ob control or ob/ob mice. Quantification of Western blots of (G) cleaved caspase 3 and (H) p-MYPT in ob control and ob/ob mice adipose tissues normalized to actin levels. Values represent mean ± S.E.M. *P < 0.05; ***P < 0.001 compared to respective controls.

To further gain insight into the potential role of adipocyte-derived MPs in recruitment of macrophages to adipose tissue during obesity, we used common genetic murine model of obesity and insulin resistance. Three-months old ob/ob mice showed a marked increase in circulating MPs compared to ob control mice (Fig 5A). Furthermore, MPs extracted from platelet free plasma (PFP) of ob/ob mice were similar to the isolated MPs from stressed 3T3-L1 adipocytes both in size (approximately 90–500 nm range; Fig 5B) and in appearance as observed by electron microscopy (Fig 5B). Since we revealed that adipocyte-derived MP release is increased in stressed adipocytes and adipocyte-derived MP were enriched in perilipin A, we next assessed for the presence of perilipin A in the circulation as a plausible indicator of AT as a source of circulating MPs. Perilipin A is known for its roles as a central gatekeeper of the adipocyte lipid storehouse and a marker of adipocyte differentiation [31, 32]. Furthermore, perilipin is not known to be a secretory protein. We observed that expression of perilipin A was detected in circulating MPs isolated from ob/ob mice (Fig 5C), showing a significant positive correlation of perilipin A levels in circulating MPs with total body weights (Fig 5D; P<0.01, R = 0.94) and epididymal fat pad weights (Fig 5E; P<0.0001, R = 0.993). These changes were associated with increased abundance of cleaved/ active caspase 3 in subcutaneous and epididymal adipose tissue of ob/ob mice compared to ob control (Fig 5F and 5G). In addition, we observed increased phosphorylated myosine phosphatase targeting protein-1 (p-MYPT1) in the same adipose tissues suggested increased Rho-associated kinase activity (Fig 5F–5H). These results suggest that adipocyte-specific MPs might be novel mediators of macrophage recruitment to adipose tissue during weight gain (S3A Fig) and adipose tissue expansion (S3B Fig) associated with macrophage infiltration (S3C Fig) and also identify potential therapeutic targets for inhibition of MP release by adipocytes.

### Transplanted MPs derived from plasma of ob/ob mouse induce monocyte activation in circulation and inflammatory macrophage infiltration to the adipocyte tissue in WT mice

**Fig 6:**
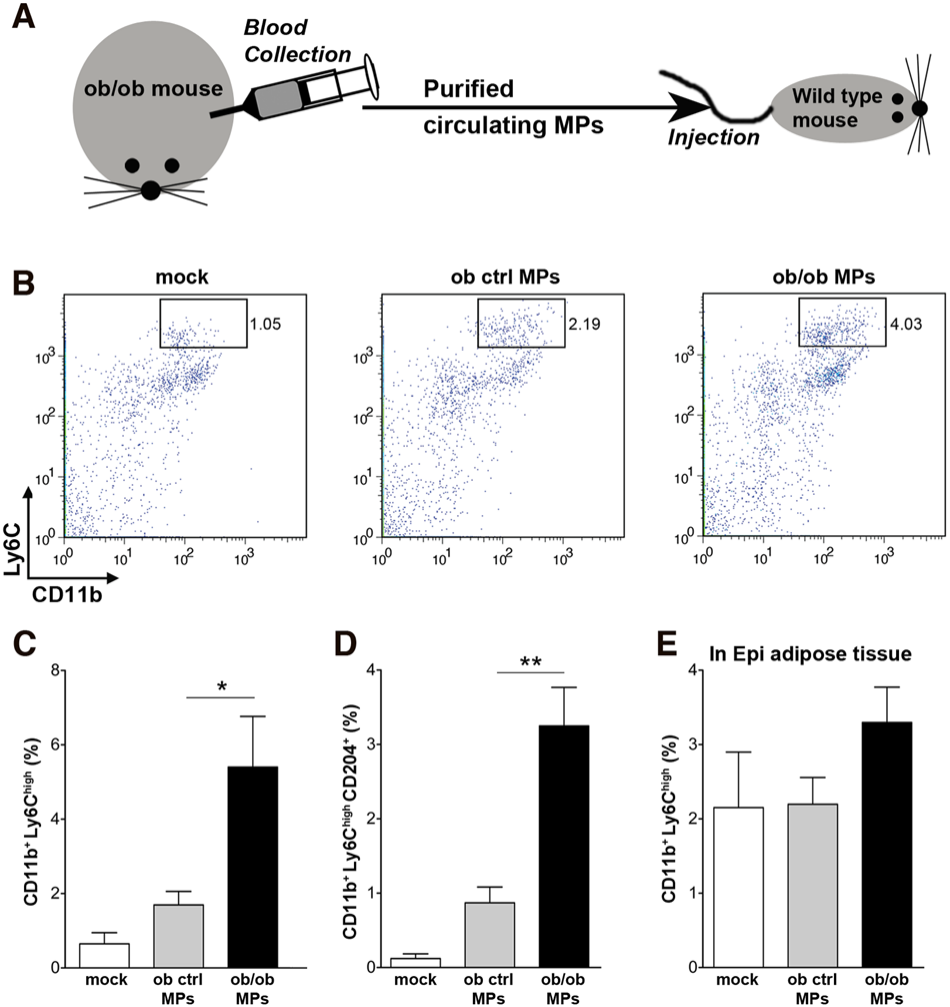
Transplanted circulating MPs from ob/ob mouse lead to monocyte activation in the blood and macrophage infiltration in the adipose tissue. (A) Scheme of transplanted experiment; ob/ob platelet free plasma was collected, circulating MPs were purified and injected into the WT mouse. (B) Dot plot analysis of the entire leukocyte population in blood resulting from the mock, ob ctrl (control) MP, and ob/ob MP injections, respectively. X-axis indicates CD11b intensity and Y-axis indicates Ly6C intensity. (C-D) Flow cytometry analysis of monocyte (CD11b^+^-Ly6C^high^) percentage (C) and activated monocyte (CD11b^+^-Ly6C^high^-CD204^+^) percentage (D) in blood resulting from the mock, ob ctrl MP, or ob/ob MP injections (n = 4 each group). (E) Flow cytometry analysis of infiltrated monocytes (CD11b^+^-Ly6C^high^) percentage in epididymal (Epi) adipose tissue from the mock, ob ctrl MP, or ob/ob MP injections. P<0.4. Values represent mean ± S.E.M. *P < 0.05; **P < 0.01 compared to ob ctrl MPs as a control.

Since we observed increased number of circulating MPs in obese mice (Fig 5A), we examined whether circulating MPs from the plasma of ob/ob mice induce chemotactic activity, such as acute inflammation and stimulation of monocyte infiltration into the adipose tissue of WT mouse. The MPs from plasma of ob/ob (ob/ob-derived MPs) or MPs from plasma of ob ctrl mice (ob ctrl-derived MPs) were purified with sucrose gradient by ultracentrifugation and injected into WT mouse (100 μg/mouse) by intravenous injection (Fig 6A). Accumulation of Inflammatory monocytes (CD11b^+^-Ly6C^high^) in the blood was three-fold higher in mice injected with ob/ob-derived MPs compared with ob ctrl-derived MPs at 6 h post injection (Fig 6B and 6C). Furthermore, there was also a three-fold increase in activated CD204^+^ (class A scavenger receptor Type I and II) monocytes in mice injected with ob/ob-derived MPs (Fig 6D). Notably, the number of infiltrated inflammatory macrophages (CD11b^+^-Ly6C^high^) to epididymal adipose tissue was also increased in the mice injected with ob/ob-derived MPs compared to ob ctrl-derived MPs (Fig 6E). These results suggest that circulating MPs in obese (ob/ob) mice have chemotactic activities for monocyte activation and infiltration to the adipose tissue.

## Discussion

The principal findings of this study relate to the mechanisms by which adipocytes induce the recruitment and migration of macrophages. The results demonstrate that stressed adipocytes release specific “find me” signals that attract monocytes and macrophages in a process that requires caspase 3 activation. We further identify adipocyte-derived MPs as the putative attraction factor both in vitro and in vivo.

Many lines of evidence have shown that a state of chronic low-grade inflammation is a key link between obesity and the associated metabolic dysregulation [4, 12, 15, 16, 33]. An important initiator of this inflammatory response is the adipose tissue, which actively secretes a variety of products such as cytokines, adipokines, and fatty acids into the circulation [10]. Macrophages, that infiltrate the adipose tissue of obese mice and humans, are the major source for some of these products, especially of pro-inflammatory cytokines such as TNF-alpha and IL-6 and chemokines such as MCP-1 [8–11, 16]. Adipose tissue macrophages has been proposed as a link between obesity and insulin resistance, however the mechanisms that initiate macrophage recruitment to adipose tissue and inflammation remains incompletely understood but it presumably involves increased secretion of chemotactic factors, in particular chemokine MCP-1, by adipose tissue [16–18]. Additionally, stressed or dying cells might display attraction signals in order to induce the migration of phagocytes [24]. Consistent with this concept, infiltrating macrophages in obese adipose tissue of mice and humans are typically clustered surrounding death adipocytes [20–22]. Yet, the molecular mechanisms linking adipocyte stress to macrophage recruitment have not been thoroughly investigated. Using an in vitro transmigration system, we showed that mature differentiated adipocytes expose to the free fatty acid, palmitic acid, a lipotoxic lipid that is abundantly present in circulation of obese individuals resulted in the release of attraction signals into their supernatants that induced migration of RAW cells and primary macrophages. While the mechanisms of removal of death cells or “eat me” signals have been extensively studied and characterized [34, 35], far less is known regarding how dying cells attract professional phagocytes to the sites of cell death, especially in adult tissues of higher organisms where phagocytes are often located at distant sites and recruited to specific tissues from the circulation [36, 37]. The secretion of soluble attracting factors from dying cells have been proposed as a tempting answer to this dilemma which could specifically direct the phagocytes to its site of action [38]. Previous studies have identified potential “find me” signals released by cancer cells and thymocytes including the nucleotides, ATP and UTP, better known as providers of metabolic energy, and the phospholipids LPC. They both had in common the need for caspase 3 activation for their release. Consistent, with these studies we found that the release of chemotactic factors by adipocytes treated palmitic acid was also mediated via activation of caspase 3. However, treatment of supernatants from adipocytes treated with palmitic acid with either apyrase or phospholipase a D, enzyme that hydrolyses nucleotides or LPC respectively failed to abolish the chemottactic activity of supernatants from dying adipocytes. Chemokines and in particular MCP-1, which are mainly produced by phagocytes themselves [9], have been proposed as potential chemotactic molecules released by adipocytes and other cell types [29, 39]. However, in our studies, the use of neutralizing anti-MCP-1 antibodies did not affect the chemotactic activity of adipocytes treated with palmitic acid. Thus, the “find me” signals released by adipocytes treated with free fatty acids appeared to be different from those reported previously. Subsequent, through several lines of evidence we were able to identify adipocyte-derived MPs, small membrane-bound particles released in a regulated manner from dying or stressed cells as a novel “find me” signal both in vivo and in vitro. The findings of the caspase 3-dependence for the production of the attraction signals in conjunction with the growing evidence that have identified MPs as key components of cell communication [40, 41] led us to the hypothesis that cell-specific-derived MPs and in particular adipocyte-derived MPs are the ideal soluble attraction signals that can travel distance in circulation and be recognized by professional phagocytes, attracting them to the specific tissue site of action. This is an issue of utmost importance, as one could envision that only “find-me” signals that can get out of the tissues and into the circulation might attract phagocytes from circulation. As of now, only short-range “find-me signals” has been described. In the case of nucleotides, they can be readily degraded by extracellular nucleotidases [42], and therefore their chemoattraction may be largely restricted to phagocytes resident within the tissue. While LPC under physiologic conditions is present in body fluids at very high concentrations and mainly bound to albumin in an inactive form incapable to bind to its receptor [35].

The release of MPs from adipocytes was both dependent of caspase 3 and Rho-associated kinase activity, which have been shown to be necessary and sufficient for the formation of membrane blebs [43] a key step in MP assembles. The release of MPs induced by palmitic acid was specific for adipocytes as undifferentiated 3T3-L1 cells or cells from the SVF exposed to this fatty acid did not show an increase release of MPs. The in vitro observations can be further extrapolated to in vivo as we found that MPs were significantly increase in the circulation from a common genetic mouse model of obesity, the ob/ob mice. These changes were associated with increased adipose tissue caspase 3 and Rho-associated kinase activity. Circulating MPs from ob/ob mice contained Perilipin A, an adipocyte specific protein suggesting adipocytes where an important source of these MPs. Moreover, we were able to show that circulating MPs from ob/ob mice were potent inducers of macrophage migration both in vitro and in vivo. Indeed in a novel approach by “transplanting” MPs from ob/ob mice into lean control mice, we were able to demonstrate that these MPs induce activation of monocytes in circulation and inflammatory macrophage infiltration to adipose tissue. Taken together, our results identify adipocyte-derived MPs as novel find me signals linking adipocyte stress to macrophage recruitment.

## Supporting Information

S1 Fig The release of “find me” signals from stressed adipocytes is not associated with changes in membrane integrity. (A) 3T3-L1 adipocytes were treated with palmitic acid, followed by flow cytometry analysis for propidium iodide staining. The percentage of necrotic cells to total parental cells analyzed by flow cytometry was assessed. (B) The assessment of cell death in 3T3-L1 adipocytes treated with palmitic acid via lactate dehydrogenase (LDH) cytotoxicity assay. Values represent mean ± S.D.
(TIF)

S2 Fig Palmitic acid exposure does not induce release of MPs from undifferentiated 3T3-L1 cells or cells from the stromal vascular fraction from adipose tissue of obese mice. (A) Number of annexin V positive mouse 3T3-L1-derived MPs assessed by flow cytometry. Values represent mean ± S.D. (B) Number of annexin V positive mouse SVF-derived MPs assessed by flow cytometry. Values represent mean ± S.D.
(TIF)

S3 Fig Body and epididymal adipose tissue gain and macrophage infiltration in ob/ob mice. (A) Body weight or (B) epididymal adipose tissue gain in ob ctrl, or ob/ob mice. (C) Flow cytometry analysis of infiltrated macrophages (F4/80^+^ or CD11c^+^) percentage in epididymal adipose tissue from the ob ctrl, or ob/ob mice. Values represent mean ± S.E.M. ***P < 0.001 compared to ob ctrl as a control.
(TIF)
