# Supplementary figures and images for: Microparticles Release by Adipocytes Act as “Find-Me” Signals to Promote Macrophage Migration
Source: PLoS One. 2015 Apr 7;10(4):e0123110. doi: 10.1371/journal.pone.0123110 (PMC4388837; doi:10.1371/journal.pone.0123110)

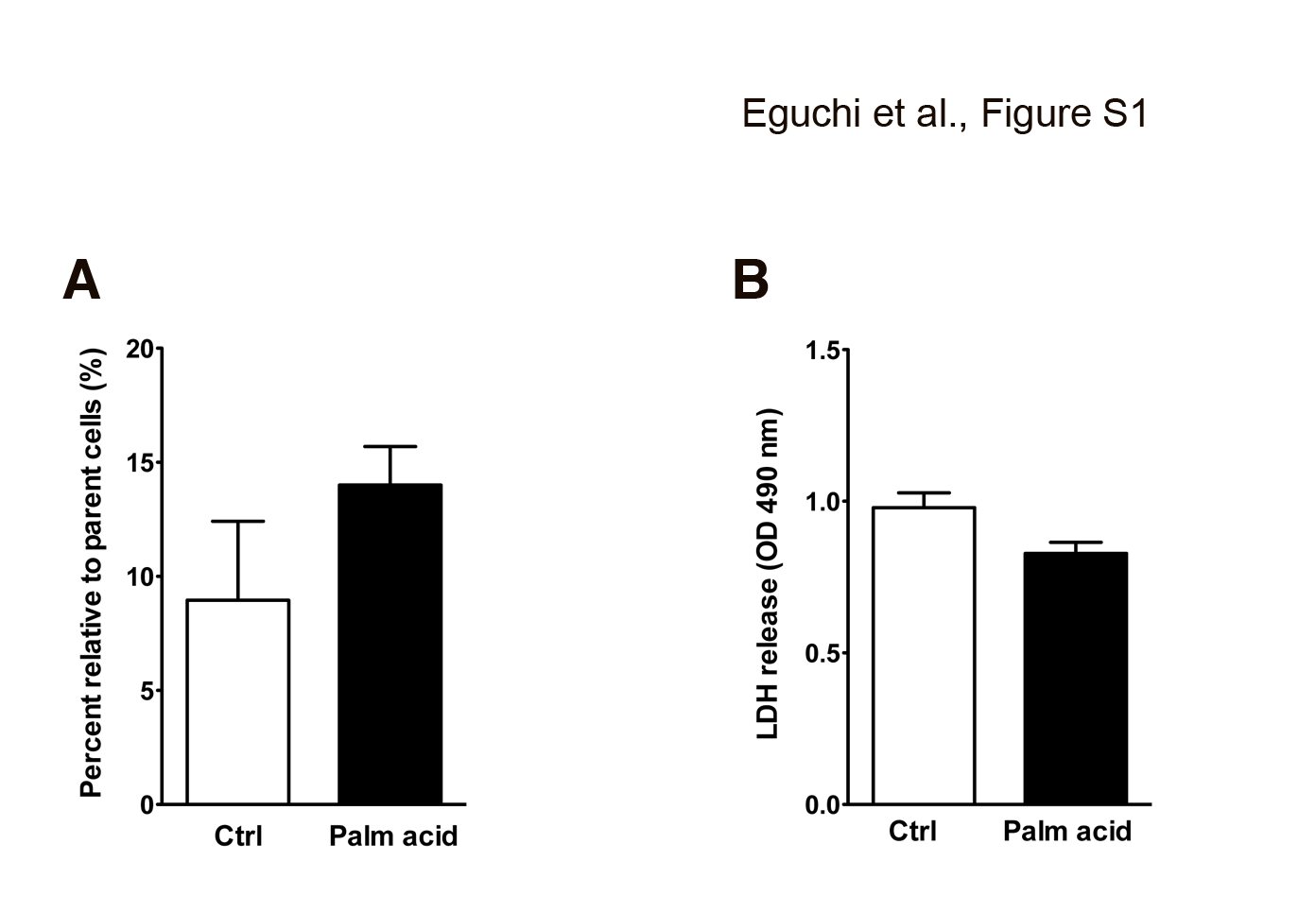

Supplement: S1 Fig — (A) 3T3-L1 adipocytes were treated with palmitic acid, followed by flow cytometry analysis for propidium iodide staining. The percentage of necrotic cells to total parental cells analyzed by flow cytometry was assessed. (B) The assessment of cell death in 3T3-L1 adipocytes treated with palmitic acid via lactate dehydrogenase (LDH) cytotoxicity assay. Values represent mean ± S.D. (TIF) [file pone.0123110.s001.tif]

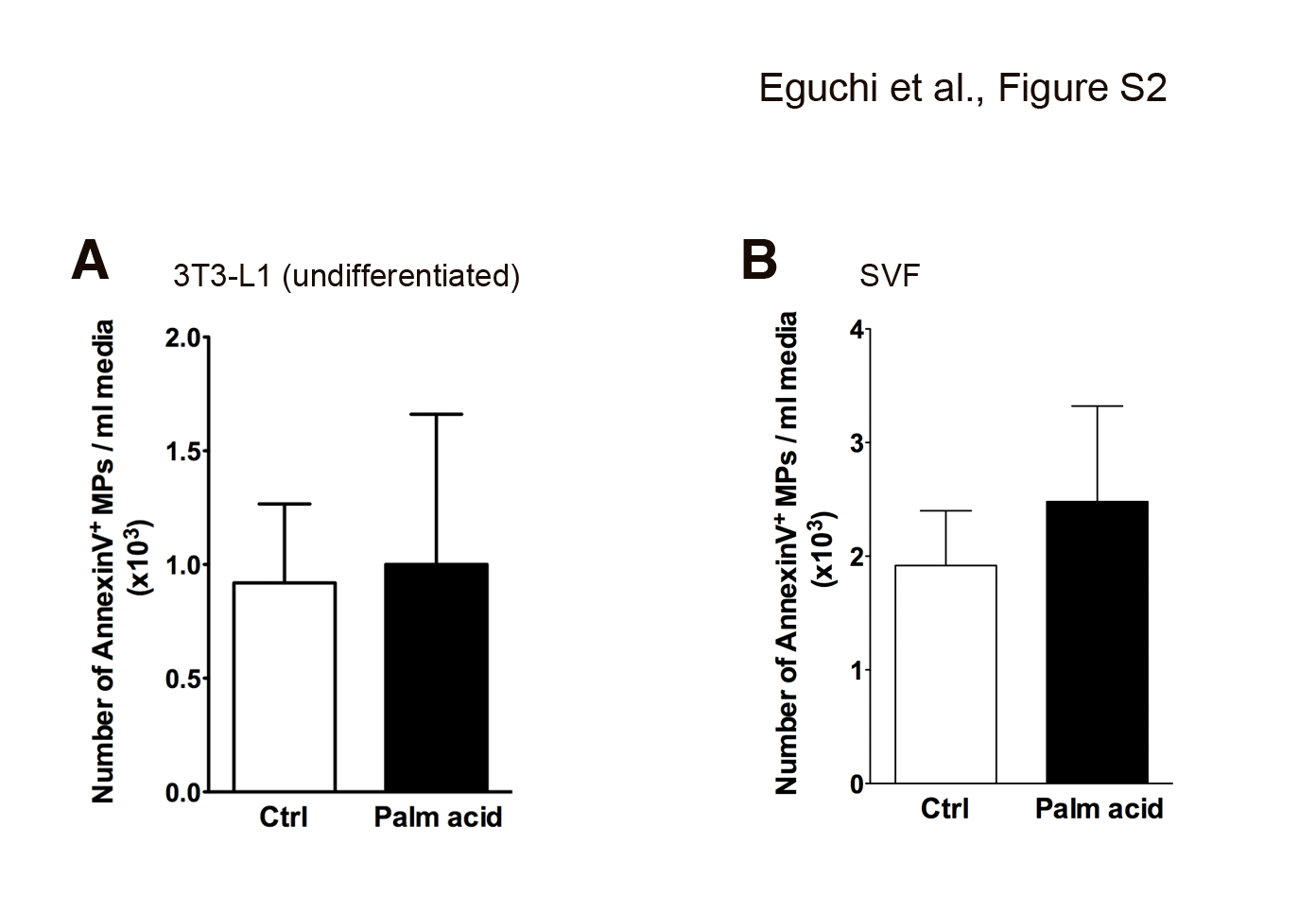

Supplement: S2 Fig — (A) Number of annexin V positive mouse 3T3-L1-derived MPs assessed by flow cytometry. Values represent mean ± S.D. (B) Number of annexin V positive mouse SVF-derived MPs assessed by flow cytometry. Values represent mean ± S.D. (TIF) [file pone.0123110.s002.tif]

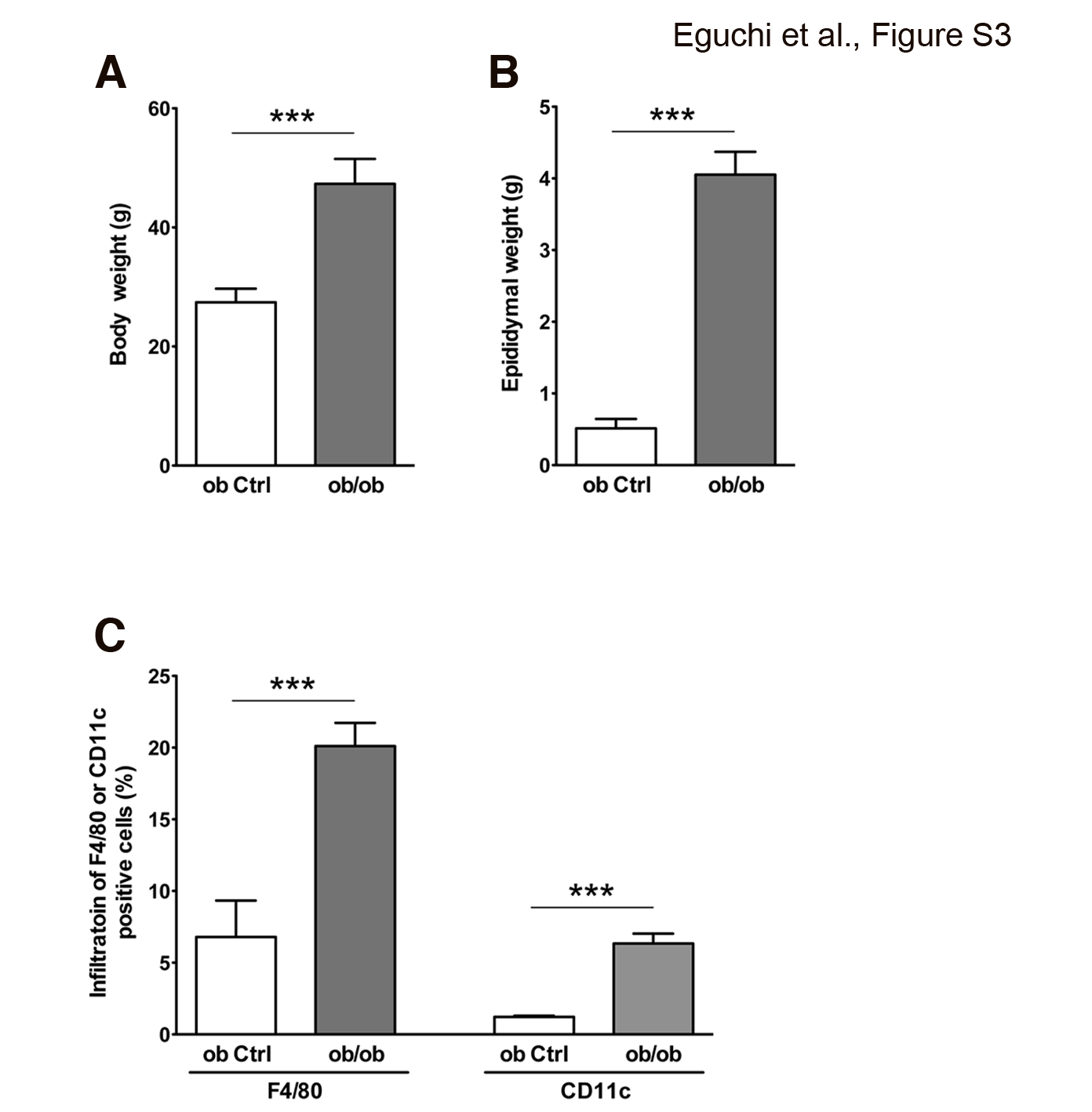

Supplement: S3 Fig — (A) Body weight or (B) epididymal adipose tissue gain in ob ctrl, or ob/ob mice. (C) Flow cytometry analysis of infiltrated macrophages (F4/80+ or CD11c+) percentage in epididymal adipose tissue from the ob ctrl, or ob/ob mice. Values represent mean ± S.E.M. ***P < 0.001 compared to ob ctrl as a control. (TIF) [file pone.0123110.s003.tif]
